# Cholecystectomy Risk in Crohn’s Disease Patients After Ileal Resection: a Long-term Nationwide Cohort Study

**DOI:** 10.1007/s11605-018-4028-y

**Published:** 2018-11-08

**Authors:** Jorn C. Goet, Evelien M. J. Beelen, Katharina E. Biermann, Annette H. Gijsbers, W. Rudolph Schouten, C. Janneke van der Woude, Annemarie C. de Vries

**Affiliations:** 1 Department of Gastroenterology and Hepatology, Erasmus University Medical Center, Rotterdam, The Netherlands; 2 Department of Pathology, Erasmus University Medical Center, Rotterdam, The Netherlands; 3 The nationwide network and registry of histopathology and cytopathology in the Netherlands (PALGA), Houten, Netherlands; 4 Department of Surgery, Erasmus University Medical Center, Rotterdam, The Netherlands

**Keywords:** Gallstones, Gallstone disease, Crohn’s disease, Inflammatory bowel disease, Cholecystectomy

## Abstract

**Background:**

The risk of gallstone disease necessitating cholecystectomy after ileal resection (IR) in Crohn’s disease (CD) patients is not well established. We studied the incidence, cumulative and relative risk of cholecystectomy after IR in CD patients, and associated risk factors.

**Methods:**

CD patients with a first IR between 1991 and 2015 were identified in PALGA, a nationwide pathology database in the Netherlands. Details on subsequent cholecystectomy and IR were recorded. Yearly cholecystectomy rates from the general Dutch population were used as a reference.

**Results:**

A cohort of 8302 (3466 (41.7%) males) CD patients after IR was identified. During the 11.9 (IQR 6.3–18.0) years median follow-up, the post-IR incidence rate of cholecystectomy was 5.2 (95% CI 3.5–6.4)/1000 persons/year. The cumulative incidence was 0.5% at 1 year, 2.4% at 5 years, 4.6% at 10 years, and 10.3% after 20 years. In multivariable analyses, female sex (HR 1.9, CI 1.5–2.3), a later calendar year of first IR (HR_/5-year increase_, HR 1.27, CI 1.18–1.35), and ileal re-resection (time-dependent HR 1.37, CI 1.06–1.77) were associated with cholecystectomy. In the last decade, cholecystectomy rates increased and were higher in our postoperative CD population than in the general population (relative incidence ratio 3.13 (CI 2.29–4.28; *p* < 0.0001) in 2015).

**Conclusions:**

Although higher in females, increasing in recent years, and higher than in the general population, the overall risk of cholecystectomy in CD patients following IR is low and routine prophylactic measures seem unwarranted.

**Electronic supplementary material:**

The online version of this article (10.1007/s11605-018-4028-y) contains supplementary material, which is available to authorized users.

## Introduction

The annual incidence of newly diagnosed gallstones in Crohn’s disease (CD) patients is twice as high as compared to the general population.^1^ Ileal disease localization and previous ileal resection (IR) have both been identified as risk factors for developing gallstones in CD patients.^1,2^ The underlying pathophysiology for the increased risk of developing gallstones in CD patients with ileal disease or after IR is not fully understood. A disturbance of the enterohepatic cycle of bilirubin, due to bile salt malabsorption in the ileum, may increase bilirubin secretion into the bile and thereby increase formation of gallstones. Alternative hypotheses are supersaturation of cholesterol in the bile due to reduced bile salt absorption or reduced motility and emptying of the gallbladder.^3–7^

Data on the prevalence of gallstones diagnosed with abdominal ultrasound in CD patients are variable, probably due to the inclusion of pooled populations of both symptomatic and asymptomatic CD patients. The reported prevalence ranges from respectively 10.4 to 38.5% in females and 9.4 to 25% in males and is clearly higher as compared to the reported prevalence in the asymptomatic general population, respectively 10.5% in females and 6.5% in males.^1,2,6,8–10^ Available epidemiological data suggests an increased risk of symptomatic and/or complicated gallstone disease in CD patients.^1,2,6,10^ A case-control study in 429 CD patients showed that the incidence rate of gallstones on abdominal ultrasound was 14.35/1000 persons/year compared to 7.48/1000 persons/year in matched hospital controls.^1^ Additionally, this study suggested a significant proportion of patients with newly diagnosed gallstones would eventually require cholecystectomy for symptomatic gallstone disease ([9/41] 22%). A major limitation of this and other reports is the inclusion of small CD populations and lack of long-term follow-up data. In order to interpret the clinical relevance of the observed increased risk of gallstones in CD patients, studies assessing the risk of gallstone disease necessitating cholecystectomy are necessary. A high risk of cholecystectomy after IR justifies increased alertness in symptomatic CD patients and possibly even prophylactic measures at the time of IR, such as synchronous cholecystectomy. In this nationwide long-term follow-up study in the Netherlands, we aimed to assess the risk of—and identify risk factors for—cholecystectomy during long-term follow-up after IR in CD patients, including absolute annual and cumulative risk as well as the relative risk as compared to the general population.

## Materials and Methods

### Histopathology Database

In the Netherlands, all histopathology and cytopathology reports are collected in the nationwide network and registry of histopathology and cytopathology in the Netherlands (PALGA). Since 1991, this database has a nationwide coverage.^11^ Every individual patient within the database is identified with a unique code that allows follow-up of all consequent pathology reports, regardless of the institute the patient is being treated. Every record in the database contains an excerpt combined with diagnostic codes given by the pathologist who assessed the tissue. The codes used are similar to the Systematized Nomenclature of Medicine (SNOMED) classification of the College of American Pathologists.^12^ After a report has been coded, it is submitted online to the central database. The current study was based on data recorded in the PALGA database between 1991 and 2015. For each patient, the following characteristics were available: gender, date of birth, date of pathology review, summary text, and diagnostic code.

### Patient Selection

All patients aged ≥ 18 years with an IR (ileal or ileocolonic resection) and simultaneous histological diagnosis CD in the period from 1991 to 2015 were identified in PALGA. Corresponding details on subsequent IR and cholecystectomy were identified using PALGA pathology codes (Appendix). Patients with a diagnosis of malignancy in the initial bowel resection specimen were excluded. Duplicate pathology reports (e.g., revision material) were excluded. Furthermore, patients with cholecystectomy prior to IR and patients of whom the first available excerpt in our database was an ileal re-resection were excluded. Patients with a cholecystectomy with gallbladder carcinoma, or gallbladder specimens resected in combination with other procedures such as hepatectomy or Whipple procedure, were excluded form analysis. Follow-up data were evaluated until December 2015. In addition, we obtained the yearly cholecystectomy rates for the general Dutch population aged ≥ 18 years from 1991 to 2015, using PALGA pathology codes, to create a reference study population (Appendix).

### Statistical Analysis

Statistical analyses were performed with IBM SPS Statistics version 22.0 (IBM Corp. Released 2013, IBM Corp, Armon, NY) and R version 3.4.0 (2017-4-21, R Foundation for Statistical Computing, Vienna, Austria) with the packages survival and splines.^13,14^ Data are presented as median and interquartile range (IQR) for continuous variables. The PALGA database does not contain follow-up data on date of death unless an autopsy has been performed. Therefore, censoring for patient death was imputed from survival data of the general Dutch population from the Statistics Netherlands agency (CBS).^15^ For each patient, the imputed follow-up was based on life expectancy in his or her year of birth, assuming the survival of CD patients is similar to that of the general population.^16,17^ Interval between IR and cholecystectomy and cumulative incidence of cholecystectomies was evaluated using Kaplan-Meier survival analysis. Univariate and multivariate Cox-regression analysis was performed to identify factors associated with cholecystectomy.

The association of a second IR with a subsequent cholecystectomy was assessed by modeling the time until a second IR as a time-dependent covariate. Accompanying Kaplan-Meier estimations were made using a clock-reset approach.^18^ In this figure, a cumulative incidence curve was plotted for patients who only had one IR. Those who had a second resection during follow-up were censored and switched to a new cumulative incidence curve starting at time 0.

In addition, we assessed the yearly crude incidence rates of cholecystectomy in our CD population as well as in the general Dutch population. Cholecystectomy rates of the general Dutch population were obtained from PALGA. Data on total population numbers over calendar years were obtained from a Dutch registry (Statistics Netherlands, www.cbs.nl).^19^ Joinpoint Regression Program, version 4.5.0.1, was used to examine significant changes in incidence over calendar time between 1991 and 2015.^20^ Relative incidence ratios (relative risk) of cholecystectomy between our CD cohort and the general Dutch population were calculated at yearly intervals for the period 2001–2015 according to Altman.^21^

This study was conducted in accordance with the protocol and the principles of the Declaration of Helsinki. The protocol was approved by the Institutional Research Board of the corresponding center.

## Results

### Baseline Cohort Characteristics

**Fig. 1:**
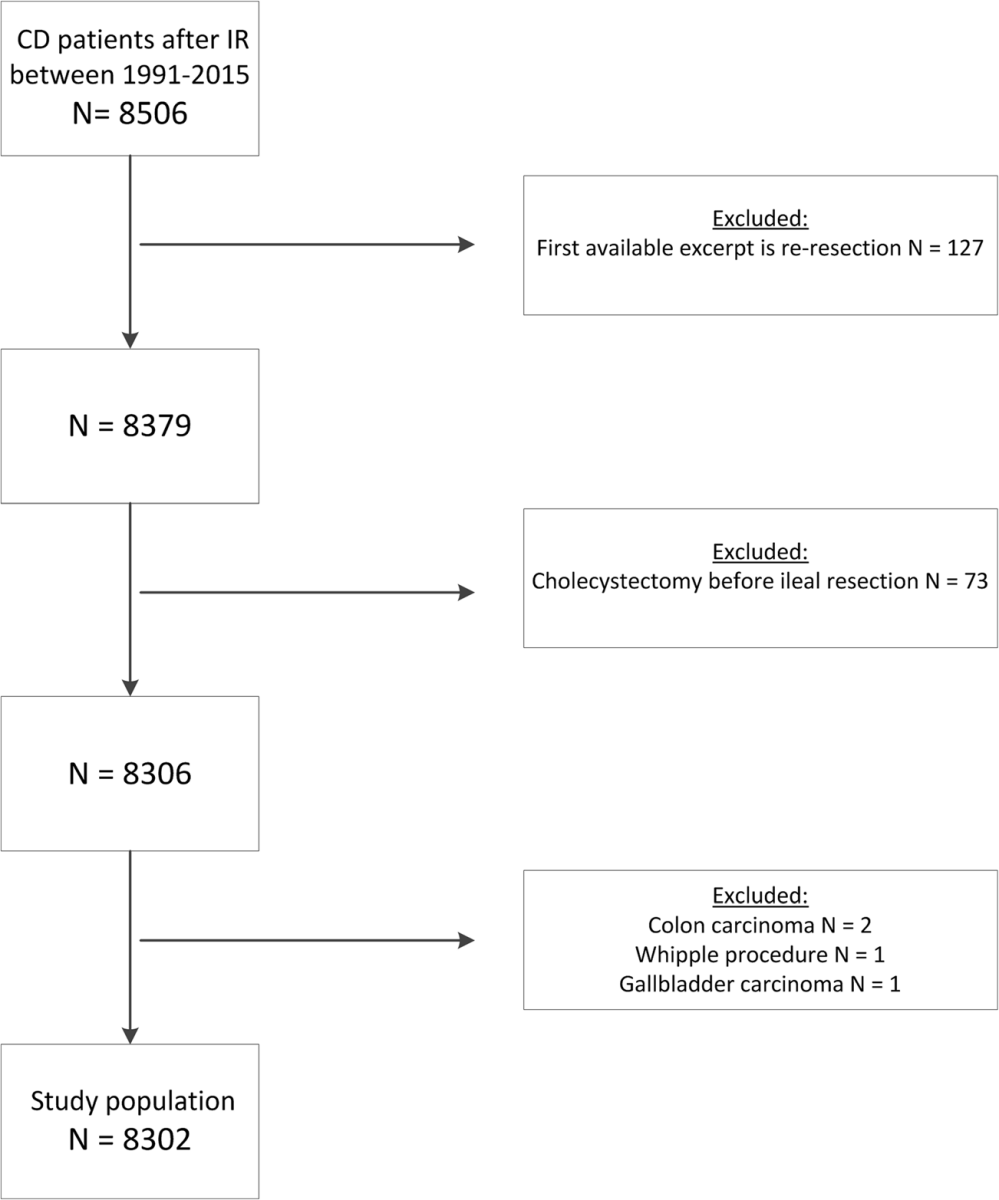
Flowchart of inclusion. CD, Crohn’s disease; IR, ileal resection

A cohort of 8506 adult CD patients who underwent an ileal resection between 1991 and 2015 was retrieved. After applying exclusion criteria, 8302 patients were included for further analysis (Fig. 1). The majority of patients was female (4836; 58.3%) and the median (IQR) age at the first resection was 37.0 (27.0–51.0) years (Table 1). Median follow-up was 11.98 (IQR 6.31–18.05; range 0.0–25.0) years.

**Table 1:** Cohort characteristics

	Study population (*N* = 8302)
Sex, male	3466 (41.7%)
Age at first IR	37.0 (22.0–51.0)
Calendar year of first resection	
1991–1995	1751 (21.1%)
1996–2000	1848 (22.3%)
2001–2005	1689 (20.3%)
2006–2010	1543 (18.6%)
2011–2015	1471 (17.7%)
Number of IR	
1 resection	7240 (87.2%)
2 resections	854 (10.3%)
> 2 resections	208 (2.5%)

Data are presented as frequency (%) or median (IQR)

*IR*, ileal resection

### Cumulative Incidence of Cholecystectomy

**Fig. 2:**
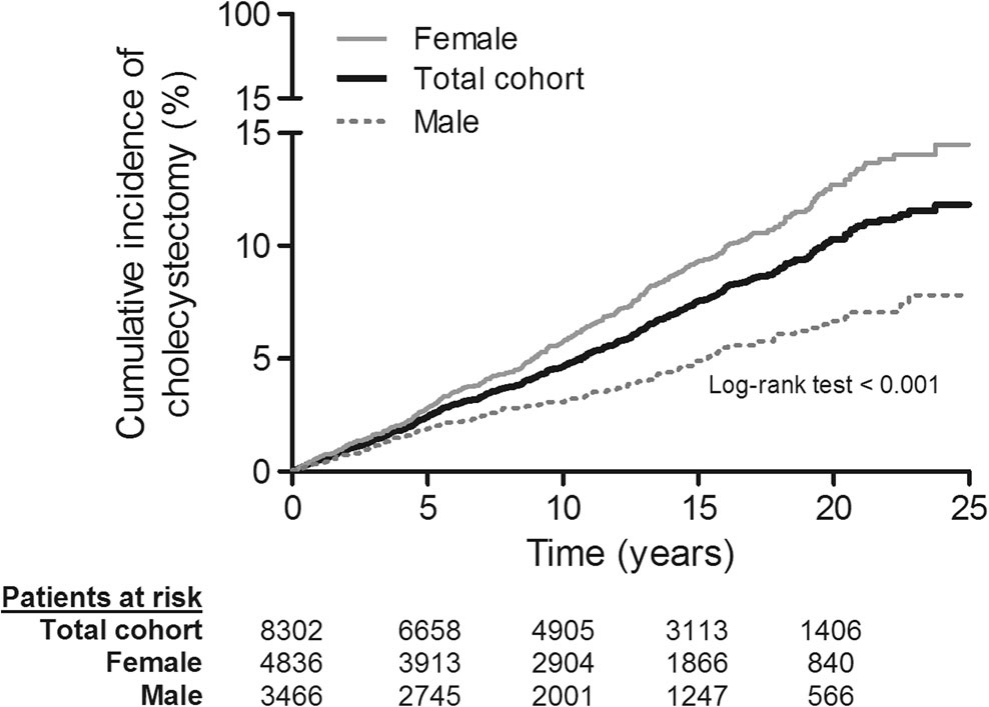
Cholecystectomy risk after ileal resection. Kaplan-Meier estimates of the occurrence of cholecystectomy in the total cohort stratified according to gender. Females had a significantly higher probability of cholecystectomy than males (log-rank test < 0.001 and HR 1.84 [95% CI 1.52–2.23; *p* < 0.001])

At the end of the 25-year follow-up period, a total of 523 (6.3%) patients had undergone a cholecystectomy: 143 males (1.7% of total population and 4.5% of male study population) and 380 females (4.6% of total population and 7.9% of female study population). The median (IQR) age at cholecystectomy was 45.65 (37.36–56.62) years. The incidence rates of cholecystectomy at 1, 5, 10, and 20 years of follow-up were 0.5%, 2.4%, 4.6%, and 10.3%, respectively (Fig. 2). Female CD patients had higher incidence rates of cholecystectomy than male patients: 0.6% vs. 0.3%, 2.8% vs. 1.9%, 5.7% vs. 3.1%, and 12.7% vs. 6.7%, at 1, 5, 10, and 20 years, respectively (Fig. 2).

### Factors Associated with Cholecystectomy

**Fig. 3:**
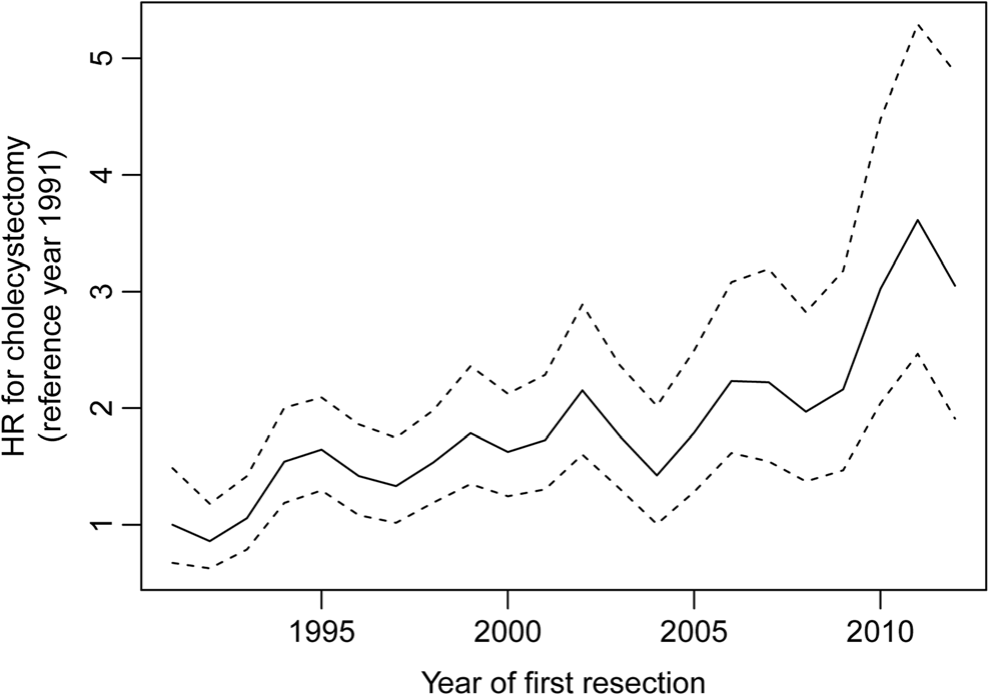
Hazard ratio of cholecystectomy over calendar year of first ileal resection. Hazard ratio (solid line) and corresponding 95% confidence interval (dashed lines) for the association between calendar year of first IR and cholecystectomy. A later calendar year of the first IR was associated with an increasing hazard for cholecystectomy

**Fig. 4:**
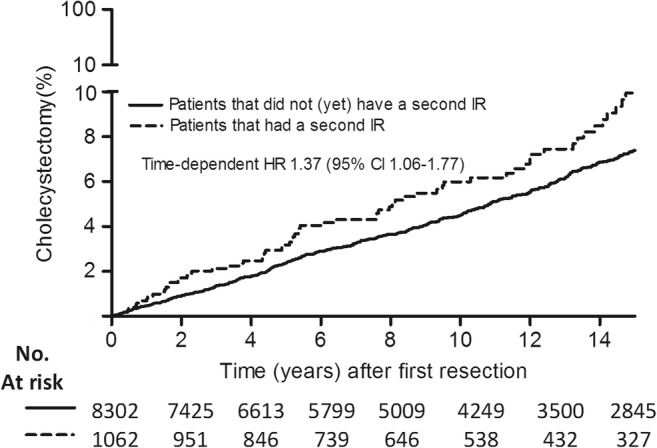
Cumulative incidence of cholecystectomy in a clock-reset approach: patients who only underwent one IR during their follow-up are in the solid line. Patients who underwent a second IR are represented in the solid line until they have a second IR. They are then censored and switched to a new survival curve (dotted line), which is then reset as time 0 for further follow-up. Patients with an ileal re-resection during follow-up had an increased probability of a cholecystectomy during their further follow-up. IR, ileal resection

In univariable analysis, female patients had a significantly higher probability of cholecystectomy than male patients (HR 1.84 (95% confidence interval [CI] 1.52–2.23); *p* < 0.001; Fig. 2). Furthermore, a later calendar year of the first IR was associated with an increased probability of cholecystectomy (HR_/5-year increase_ 1.25; CI 1.16–1.34; Fig. 3). Finally, ileal re-resection during follow-up was associated with a slightly increased probability of cholecystectomy (time-dependent HR 1.30; CI 1.01–1.68; *p* = 0.045; Fig. 4). All these variables remained significantly associated with a cholecystectomy in multivariable analysis (Table 2).

**Table 2:** Covariates associated with cholecystectomy

	Univariable analyses			Multivariable analyses		
	HR	95% CI	*p*	HR	95% CI	*p*
Female sex	1.839	1.517–2.229	< 0.001	1.856	1.532–2.250	< 0.001
Age at first resection	1.000	0.994–1.005	0.920			
Year of first IR, per 5 years	1.252	1.164–1.341	< 0.001	1.265	1.177–1.350	< 0.001
Ileal re-resection during FU^a^	1.299	1.006–1.678	0.045	1.369	1.059–1.769	0.016

^a^These hazard ratios were obtained by considering re-resection as a time-dependent covariate in univariable and multivariable analyses

*HR*, hazard ratio; *CI*, confidence interval; *IR*, ileal resection; *FU*, follow-up

### Yearly Cholecystectomy Rates in CD Patients and the General Population

**Fig. 5:**
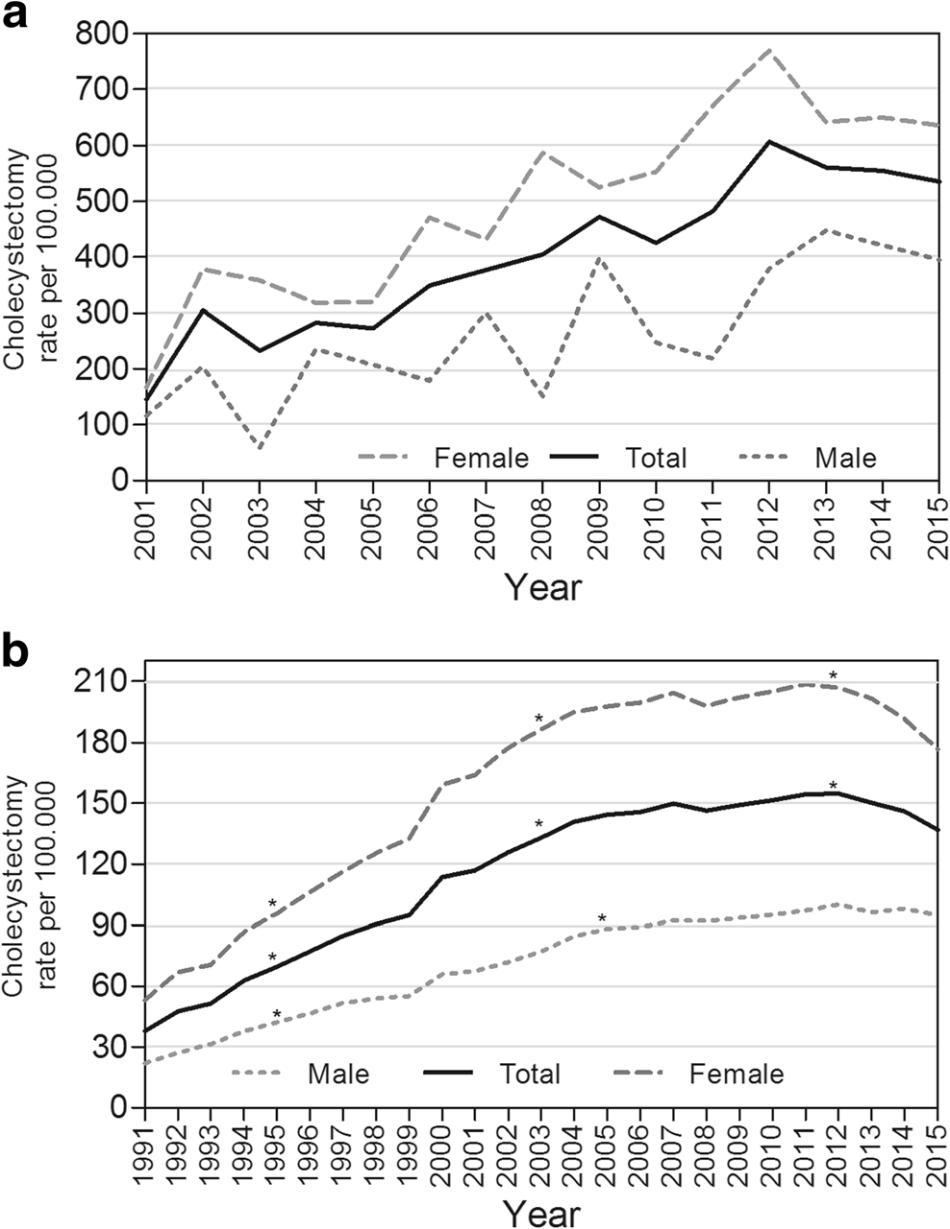
**a** Incidence rates of cholecystectomy per calendar year in CD patients. Crude incidence rates of cholecystectomy increased in our postoperative CD population from 2001 to 2015. Cholecystectomy rates between 1991 and 2001 are not presented in this figure because these initial years may not be representative as CD patients who underwent IR before 1991 are not included as background population. **b** Incidence rates of cholecystectomy in the general Dutch population per calendar year. Crude incidence rates of cholecystectomy increase over calendar year in the general Dutch population in males and females. The asterisk indicates joint points where the annual percentage change (APC) is significantly different from 0 at the alpha = 0.05 level, indicating a significant trend

During the median follow-up of 12 years, the incidence rate of cholecystectomy in our CD cohort was 5.2 (95% CI 3.5–6.4)/1000 persons/year. Females had a significantly higher rate than males (6.4 [95% CI 4.7–5.6] vs. 3.5 [95% CI 2.9–4.1]/1000 persons/year; *p* < 0.0001). Absolute cholecystectomy rates per calendar year were higher in our postoperative CD population (Fig. 5a) than in the general Dutch population (Fig. 5b) between 1991 and 2015. Over the last decade, the relative incidence ratio for our postoperative CD cohort, compared to the general population, varied between 1.28 (95% CI 1.28–2.81; *p* = 0.001) in 2002 and 3.13 (95% CI 2.29–4.28; *p* < 0.0001) in 2015. For males, these ratios were 2.16 (95% CI 1.028–4.52; *p* = 0.042) and 3.25 (95% CI 1.85–5.72; *p* < 0.0001) and for females 1.68 (95% CI 1.06–2.67; *p* = 0.027) and 2.90 (1.99–4.22; *p* < 0.0001), respectively (Supplementary table 1). In accordance with the observed increase in the probability of cholecystectomy after IR in our CD population over calendar time, there was an increase in the crude incidence rates of cholecystectomies within the study population (Fig. 5a), which was also observed in the general Dutch population with a significant increase over calendar time from 48/100,000 in 1991 to 185/100,000 in 2006, and remained relatively stable with a minimal decline after 2012 (Fig. 5b).

## Discussion

It has been well established that CD patients are at an increased risk of gallstone development, especially those with ileal involvement. The clinical relevance of this observed increase has however remained unclear. This large nationwide long-term follow-up study is the first to assess the risk of gallstone disease necessitating an intervention following IR, namely cholecystectomy. Our results show that, although over the past years the incidence of cholecystectomy in post-IR CD patients has increased and is currently higher than that in the general population, the annual incidence of cholecystectomy after IR is low. With thorough analyses, we were able to identify patients more likely to require cholecystectomy following IR. Female patients, those undergoing ileal re-resection, and patients with a later calendar year of first IR have an increased probability of cholecystectomy.

The observed incidence rate of cholecystectomy in our CD population after IR of 5.2/1000 persons/year is evidently lower than the reported incidence rate of gallstones found by ultrasound examination in CD patients in general. A large case-control study in 429 CD patients reported an incidence rate of gallstones on abdominal ultrasound of 14.35/1000 persons/year, which was significantly higher than that in matched controls (7.75/1000 persons/year, *p* = 0.012).^1^ Additionally, it was shown that in a subgroup of CD patients with newly developed gallstones, about 22% of the patients (9/41) eventually required cholecystectomy for symptomatic stones. Our nationwide study substantially adds to these data by describing the clinical consequences of gallstones in a large long-term follow-up cohort of CD patients. Our study shows that in a potentially high-risk population for gallstone disease, only a minority of patients of approximately 10.5% during 20 years will eventually require a cholecystectomy.

The observed cumulative incidence of cholecystectomy of 4.6% within 15 years of follow-up in our study is evidently higher than that observed in the general population. A recent large population-based cohort study of over 65,000 individuals found a cumulative incidence of cholecystectomy for gallstone disease of 1.8% within 15 years of follow-up.^22^ To date, the only published study on gallstone disease necessitating cholecystectomy in CD patients was a case-control study including 134 CD patients with ileitis.^10^ This study demonstrated that the incidence of cholecystectomy was not significantly different from an age- and sex-matched control group. The nationwide data in the current study significantly adds to previous reports by quantifying the rate of cholecystectomy in absolute and relative risk, annual cholecystectomy risk, and cumulative cholecystectomy risk during long-term follow-up.

Female sex is an important risk factor for cholecystectomy after IR in our study. This finding is in agreement with data from the general population, in which female sex has been identified as an important risk factor for gallstone development.^9,23^ Especially women in fertile years are more likely to form gallstones as compared to men. This difference narrows after menopause.^24,25^ However, previous data on the prevalence of gallstones in CD patients indicate there are no gender differences. A study in 251 CD patients showed no gender differences in the prevalence of gallstones (27% in females vs. 29% in males).^2^ In accord, a more recent study in 330 CD patients reported similar results with a prevalence of 25% in females vs. 25% in males as found by ultrasound examination.^6^ A possible explanation for the observed difference in our cohort may be the long-term follow-up and the larger number of included CD patients. Alternatively, the difference may be explained by our cohort’s relatively young median age of 37 years.

In line with expectations, ileal re-resection was associated with cholecystectomy. Previous studies have shown that the prevalence of gallstones is associated with the number of bowel resections and is significantly increased in patients in whom more than 10 cm of the ileum was resected.^2,26^ In the current study, we assessed re-resection as a surrogate for the length of intestine removed by surgery and/or CD severity, and this was associated with an increased risk of cholecystectomy.

To our knowledge, this is the first study to report on an increase in gallstone disease necessitating cholecystectomy after IR in CD patients over calendar time. Our findings may well reflect the global trend of increasing gallstone prevalence observed in necroptic^27^ and ultrasound studies.^24,28^ In addition, cholecystectomy rates have increased, especially in the first decade after 1990.^29–31^ This increase may be attributed to the introduction of laparoscopic cholecystectomy in 1990, which may have lowered the threshold for a surgical procedure in cases of uncomplicated gallstones.^32,33^ Still, the cholecystectomy rates may vary greatly between different countries. A recent nationwide study from Sweden, which assessed a total of 130,800 laparoscopic and 47,641 open cholecystectomies performed between 1998 and 2013, showed the annual rates of cholecystectomies remained stable.^34^ In contrast, our data covering all cholecystectomies performed within the Netherlands between 1991 and 2015 indicates annual rates have increased between 1991 and 2007 and remained relatively stable with a minimal decline after 2012. This corresponds with the observed increase in probability of cholecystectomy after IR in our study population.

One of the strengths of our study is the selection of a large nationwide cohort with long-term follow-up data, with stringent inclusion criteria of a histology-proven CD and the use of the general Dutch population as a reference population. These data allowed a thorough assessment of gallstone disease necessitating cholecystectomy including risk factors, absolute, relative, and cumulative risk. In addition, due to the nationwide coverage of PALGA, there was a long-term follow-up for each individual patient after IR and full-scale cholecystectomy detection. However, some limitations need to be considered. Firstly, our inclusion criteria for a biopsy-proven CD (SNOMED D62160) might have been too stringent as some pathology reports only include SNOMED codes for ulcer, granuloma, or inflammation. Secondly, the PALGA database provides limited data on patient characteristics, making further subgroup analysis impossible. No data on initial diagnosis of CD and thus duration and/or severity of disease, length of the resected segment, ileal involvement earlier in the disease course, or other known risk factors (e.g., BMI, bariatric surgery) are provided. It would be highly interesting to assess these factors in such a large cohort. In addition, the PALGA database does not contain a date of CD diagnosis, thereby limiting the possibilities for time-to-event and risk factor analysis in a cohort of CD patients without IR. Further studies could focus on the comparison of the risk of cholecystectomy in CD patients without IR and those with IR. This would provide further evidence for the hypothesized role of IR in the development of gallstones in CD patients. Finally, data on the indication of cholecystectomy are lacking. Cholecystectomy rates in CD patients might be higher due to frequent contact with health care professionals and consequently a lower threshold for performing an abdominal ultrasound and a higher diagnosis rate of incidental gallstones. Furthermore, cholecystectomy may have been performed for indications other than complications of chole(cysto)lithiasis, e.g., polyp, tumor-like lesions, acalculous cholecystitis, and unexplained abdominal pain. However, in general, only a small proportion of patients undergo cholecystectomy for these indications.^34^

In conclusion, this large nationwide study shows that annual incidence of cholecystectomy in CD patients after IR is 0.5% and increases almost linearly during follow-up to 10.5% after 20 years. Female sex, re-resection, and a later year of IR are associated with cholecystectomy. The incidence of cholecystectomy after IR in CD patients is currently three times higher than that in the general population. Nonetheless, overall, the risk of cholecystectomy is low. While this risk may justify increased alertness of gallstone disease and a lower threshold for abdominal ultrasound in symptomatic CD patients following ileal resection, it does not seem to warrant prophylactic synchronous cholecystectomy during IR in all CD patients.

### Electronic Supplementary Material

ESM 1 (DOCX 17 kb)

## Appendix

### PALGA diagnosis codes used in the analysis of our CD cohort

Crohn’s disease: D62160

Ileum or ileocecal: T65200, T65500T67000, T65500, M81406

Resection: P11100, P11200, P11101

Gallbladder: T57000

Adenocarcinoma M81403

Metastasis, adenocarcinoma M81406

Metastasis, carcinoma M80106

Intramucosal carcinoma M80105

### PALGA codes used to extract cholecystectomy rates in general Dutch population

Cholecystectomy: T57000111000

Gallbladder and excision: T57___ AND *54*

## Abbreviations

CD: Crohn’s disease
IR: Ileal resection
HR: Hazard ratio
RR: Relative incidence ratio
CI: Confidence interval
