# Supplementary material for: Cholecystectomy Risk in Crohn’s Disease Patients After Ileal Resection: a Long-term Nationwide Cohort Study
Source: J Gastrointest Surg. 2018 Nov 8;23(9):1840–7. doi: 10.1007/s11605-018-4028-y (PMC6702183; doi:10.1007/s11605-018-4028-y)
Supplement: Supplementary file 1 — (DOCX 17 kb) [file 11605_2018_4028_MOESM1_ESM.docx]

| **Calendar** | **Total cohort** | | | **Female** | | | **Male** | | |
| --- | --- | --- | --- | --- | --- | --- | --- | --- | --- |
| **Year^a^** | **RR** | **95% CI** | **p** | **RR** | **95% CI** | **p** | **RR** | **95% CI** | **p** |
| 2001 | 0.98 | 0.55-1.72 | 0.9359 | 0.81 | 0.40-1.61 | 0.5419 | 1.31 | 0.49-3.49 | 0.5903 |
| 2002 | 1.90 | 1.28-2.81 | 0.0014 | 1.68 | 1.06-2.67 | 0.0270 | 2.16 | 1.03-4.52 | 0.0420 |
| 2003 | 1.37 | 0.87-2.15 | 0.1707 | 1.52 | 0.94-2.44 | 0.0846 | 0.58 | 0.14-2.31 | 0.7790 |
| 2004 | 1.57 | 1.05-2.37 | 0.0293 | 1.29 | 0.78-2.14 | 0.3217 | 2.12 | 1.06-4.24 | 0.0335 |
| 2005 | 1.48 | 0.98-2.25 | 0.0651 | 1.28 | 0.77-2.12 | 0.3376 | 1.79 | 0.85-3.75 | 0.1235 |
| 2006 | 1.88 | 1.30-2.73 | 0.0008 | 1.87 | 1.23-2.84 | 0.0032 | 1.54 | 0.69-3.42 | 0.2923 |
| 2007 | 1.98 | 1.38-2.83 | <0.0001 | 1.68 | 1.09-2.60 | 0.0200 | 2.48 | 1.34-4.61 | 0.0040 |
| 2008 | 2.18 | 1.54-3.09 | <0.0001 | 2.36 | 1.62-3.44 | <0.0001 | 1.26 | 0.52-3.03 | 0.5180 |
| 2009 | 2.50 | 1.81-3.45 | <0.0001 | 2.08 | 1.39-3.10 | <0.0001 | 3.27 | 1.90-5.62 | <0.0001 |
| 2010 | 2.23 | 1.58-3.13 | <0.0001 | 2.16 | 1.46-3.19 | <0.0001 | 2.00 | 1.00-4.00 | 0.0494 |
| 2011 | 2.49 | 1.80-3.43 | <0.0001 | 2.58 | 1.81-3.69 | <0.0001 | 1.74 | 0.83-3.65 | 0.1421 |
| 2012 | 3.12 | 2.34-4.16 | <0.0001 | 3.00 | 2.14-4.19 | <0.0001 | 2.93 | 1.66-5.15 | 0.0002 |
| 2013 | 2.98 | 2.21-4.03 | <0.0001 | 2.57 | 1.78-3.72 | <0.0001 | 3.61 | 2.14-6.09 | <0.0001 |
| 2014 | 3.04 | 2.24-4.13 | <0.0001 | 2.75 | 1.90-3.97 | <0.0001 | 3.35 | 1.94-5.76 | <0.0001 |
| 2015 | 3.13 | 2.29-4.28 | <0.0001 | 2.90 | 1.99-4.23 | <0.0001 | 3.25 | 1.85-5.72 | <0.0001 |

**Supplementary table 1.** Relative incidence ratios between Crohn’s disease patients and the general Dutch population between 2001 and 2015.

RR, relative incidence ratio; CI, confidence interval.

^a^ Relative incidence ratios could not be calculated for the period 1991-2000 because of the low number of events in this time period.
